# Investigation of Anxiety, Depression, Sleep, and Family Function in Caregivers of Children With Epilepsy

**DOI:** 10.3389/fneur.2021.744017

**Published:** 2021-10-25

**Authors:** Meiyan Zhang, Huiting Zhang, Shuxian Hu, Man Zhang, Yu Fang, Junjie Hu, Jianxiang Liao

**Affiliations:** ^1^Center of Child Healthcare and Mental Health, Shenzhen Children's Hospital, Shenzhen, China; ^2^China Medical University, Shenzhen Children's Hospital, Shenzhen, China; ^3^Department of Neurology, Shenzhen Children's Hospital, Shenzhen, China; ^4^Shenzhen Children's Hospital Affiliated to Medical College of Shantou University, Shenzhen, China

**Keywords:** children with epilepsy, caregiver, depression, anxiety, sleep, family function

## Abstract

**Objective:** Epilepsy is a chronic disease that places a heavy burden on caregivers. Previous studies have shown that caregivers of epilepsy patients often experience anxiety and depression; however, few comprehensive studies have assessed their sleep quality and family function. Based on the current understanding of the anxiety and depression state of caregivers in children with epilepsy, we further explored the caregivers' sleep and family function and evaluated the predictors of the depression state of caregivers.

**Methods:** In this cross-sectional online anonymous survey, we sent an online questionnaire to the caregivers of children with epilepsy who visited our hospital. The QR code of the questionnaire was scanned at the follow-up course to conduct an online survey. The questionnaire contained questions about sociodemographic and clinical information, the Self-rating Anxiety Scale, Self-rating Depression Scale, Pittsburgh Sleep Quality Index, and the Family Assessment Device.

**Results:** A total of 308 caregivers of children with epilepsy aged 0–12 years were included in this study. The mean age of children with epilepsy was 4.8 ± 3.18 years, and the average illness duration was 34.2 ± 29.18 months. Further, 47.1% of the children took three or more anti-seizure medications, and 43.2% were on ketogenic diet therapy. We found that in 77.9% of the cases, the subjects were the mothers, in 89% there was more than one co-caregiver, and in 51.9%, financial help was required. Further, 63.6% of the caregivers thought they could not get enough access to disease knowledge education, and 83.7% perceived epilepsy was a terrible disease. Our results also showed that 65.6% of the caregivers were in depression status, 41.9% were in anxiety status, and 49.0% had poor sleep quality. The proportion of unhealthy family functioning in each subscale was 45.1–96.1%, and the unhealthy behavior control function accounted for 96.1%. Binary logistic regression analysis of the data showed that without co-caregivers [odds ratio (OR), 5.193], free of anxiety status (OR, 0.063), good sleep quality (OR, 0.446), healthy family role dimension (OR, 0.344), and healthy family general functional dimension (OR, 0.259) were predictors of depression status in caregivers of children with epilepsy.

**Conclusion:** Anxiety and depression status are common in caregivers of children with epilepsy, with depression status being more prominent. Moreover, a considerable proportion of caregivers had poor sleep quality and unhealthy family function. The caregivers' anxiety status, sleep quality, family role dimension, family general function dimension, and the number of co-caregivers were predictors of depression status in caregivers. In clinical practice, caregivers' anxiety and depression status, poor sleep quality, and unhealthy family functioning should be addressed along with the treatment of children with epilepsy.

## Introduction

Epilepsy is a common chronic neurological disorder in children, with a prevalence of 3.2–5.5‰ in developed countries, 3.9–44‰ in developing countries (1), and 3.9–5.1‰ in China (2). Epilepsy in children is often associated with developmental abnormalities in the brain, leading to the impaired motor, perceptual, and cognitive development (3, 4). As children develop physically and psychologically, sick children often develop psychobehavioral abnormalities, such as attention-deficit/hyperactivity disorder, autism, and mood disorders that affect the child's education, future work, and marital status (5). In addition to comprehensive care for the child, caregivers of children with epilepsy also need to deal with developmental problems, educational and marital problems, and the stigma caused by the disease (6). It has been shown that most parents of children with epilepsy have negative perceptions of others' reactions (53.3%). They often experienced a sense of shame, self-blame, fear, anxiety, and depression. Parents often feared divulging their child's epilepsy to their friends and relatives. And they also limit family's social interaction, which also increased their parenting pressure (7).

Anxiety and depression are common among caregivers of children with epilepsy, and previous studies reported proportion of depressive symptoms ranging 21.6–34.9%, anxiety status in 14.5% of caregivers, and poor sleep quality in 37.6% of caregivers (8–11). A study that evaluated the impact of severe pediatric epilepsy on experienced stress and psychopathology in parents in Denmark showed that among 152 respondents, the incidence of depression was 34.9%, of which 15.8% had severe depressive symptoms and 19% had moderate depressive symptoms; 14.5% of the parents were in a state of anxiety (8).

In China, a survey conducted by Peking Union Medical College Hospital in 2009 showed that compared with the healthy control group, parents of children with epilepsy had more severe anxiety and depression, which were related to low quality of life (9). A recent study in Hunan Province, China, assessed the sleep quality, anxiety, and depression of 234 parents of children with epilepsy and 230 parents of healthy children; 23.51% of the parents of children with epilepsy had depressive symptoms. The symptoms of anxiety and depression were more severe than those of healthy children, and sleep quality was worse (10). A study in Shandong Province, China, also showed that 21.6% of the parents of children with epilepsy had moderate to severe depressive symptoms, and 37.6% had poor sleep quality (11).

As mentioned previously, epilepsy caregivers often suffered from anxiety, depression, and poor sleep quality. Studies suggested that seizures' frequency, severity, and unpredictability compose of epilepsy burden on families (12). The main influencing factors of caregivers' stress are the child's emotional–behavioral problems and social difficulties, caregivers' control over their own situation, social support (8), and family resilience (11).

Based on an investigation of the anxiety and depression status of caregivers in children with epilepsy, this study further explored caregivers' sleep quality and family function and evaluated the predictors of depression status in caregivers. It was the first time to evaluate the caregiver's psychological status comprehensively from different dimensions. Then, we could take more accurate and targeted measures to help caregivers adjust their own state in order to improve caregivers' ability of raising children with epilepsy. This study assumed that abnormal emotion was common in epileptic caregivers, highly related to the disease state of epileptic children, caregivers' own condition, and family support.

## Materials and Methods

This was a cross-sectional anonymous web-based study from April 2021 to May 2021. The recruitment was performed using convenience sampling. The invitation links to online questionnaires were sent via the medical record follow-up system. Caregivers of children with epilepsy at the hospital also could complete the questionnaire online by scanning the QR code of the questionnaire directly.

### Participants

Inclusion criteria were as follows: caregivers of children with epilepsy aged 0–12 years, children with a diagnosis of epilepsy certified by neurologists, and were treated in the Epilepsy Center of Shenzhen Children's Hospital, and caregivers who agreed to cooperate with online surveys.

Caregivers who refused or were unable to provide consent or were illiterate or unable to read and fill in the questionnaire were excluded. Children with epilepsy coexisting with other chronic diseases (i.e., heart disease, tumors, leukemia, congenital heart disease, diabetes), which could affect the family function and mental states of caregivers, were excluded from this study.

A total of 308 caregivers were enrolled in the study, including 65 fathers and 240 mothers, and 308 children were involved. The average age of children with epilepsy was 4.8 ± 3.18 years, with the proportions of the age groups 0–3, 3–6, and 6–12 years being almost the same, with boys and non–only children being more common.

### Instrument

#### General Information

A self-made questionnaire was used to collect sociodemographic and clinical information such as disease status of children with epilepsy, employment status of caregivers, family income, caregivers' view of the disease, ability to pay for the disease, and so on.

#### Self-Rating Depression Scale

It is a 20-item scale to evaluate the presence and degree of the status of depression in adults within the past week. Each of the 20 items is given a severity score from 1 (none or a little of the time) to 4 (most of or all the time), but 10 items need to be scored in reverse. The raw scores obtained on the 20 items range from 20 to 80, and the standard score is converted to a whole number that ranges from 25 to 100. As the score rises, the severity of depression increases (13). The scale has been widely used in diagnostic evaluation, curative effect evaluation, and epidemiological investigation since it was introduced to China in 1985. In the Chinese norm, depression status is considered when the standard score was more than 53 within the past week (14).

#### Self-Rating Anxiety Scale

Similarly to Self-rating Depression Scale, the Self-rating Anxiety Scale (SAS) is a 20-item scale to evaluate the presence and degree of anxiety status in adults within the past week. Each of the 20 items is given a severity score from 1 (none or a little of the time) to 4 (most or all of the time), and 5 items need to be scored in reverse. The raw score obtained on the 20 items ranges from 20 to 80, and the standard score is converted to a whole number that ranges from 25 to 100. As the score rises, the severity of anxiety increases (13). A standard score of more than 50 (Chinese norm) in SAS is considered as anxiety status (14).

#### Pittsburgh Sleep Quality Index

The Pittsburgh Sleep Quality Index is a self-rated questionnaire that assesses sleep quality and disturbances over a 1-month time interval. Eighteen self-assessment items generate seven “component” scores: subjective sleep quality, sleep latency, sleep duration, habitual sleep efficiency, sleep disturbances, use of sleeping medication, and daytime dysfunction. The sum of scores for these seven components yields one global score. Each component score ranges from 0 to 3, and the global score ranges from 0 to 21. The higher the score, the worse the sleep quality (15). Xianchen et al. had proved the applicability of the Chinese version in 1996. This scale is widely used to evaluate sleep quality in clinical practice and research. The cutoff scores of the poor sleep quality is 7 in the Chinese norm (16).

#### The Family Assessment Device

The Family Assessment Device (FAD) is a 60-item questionnaire based on the McMaster model, which measures family functioning. FAD includes seven dimensions: problem solving, communication, roles, affective responsiveness, affective involvement, behavior control, and general functioning. Higher scores indicate worse family functioning. Clinical cutoff scores were used to distinguish between healthy and unhealthy family functioning on each dimension. The cutoff scores of problem solving, communication, roles, affective responsiveness, affective involvement, behavior control, and general functioning were 2.2, 2.2, 2.3, 2.2, 2.1, 1.9, and 2.0. The Chinese cutoff score of FAD has not yet been established. Cutoff scores above were used in this study to differentiate healthy and unhealthy family functioning on each dimension (14).

### Statistical Analysis

Statistical analysis using IBM SPSS Statistics software (version 23.0) was performed with a significance level defined at 0.05. All demographic data were analyzed descriptively, with nominal data presented as frequencies and percentages and continuous data presented as means and standard variations [mean ± standard deviation (SD)].

First, the data were extracted and transferred to standard data from the detailed interview in the first part of the questionnaire. Descriptive statistics were applied to analyze the sociodemographic and clinical information of children with epilepsy and caregivers, mood, sleep, and family function of caregivers. The means, SDs, and frequencies were calculated. Second, the caregivers of children with epilepsy were divided into two groups according to whether they had depression status or not. The proportion of each group in variables was tested by the χ^2^ test. Third, based on the result of the previous χ^2^ test, variables with significant differences were selected. Logistic regression analysis was carried out to identify the factors that predicted depression status in caregivers of children with epilepsy.

### Ethics Approval

The study was approved by the Shenzhen Children Hospital Ethics Committee (ethics approval no.: 2021079).

## Results

### Sociodemographic and Clinical Information of Epileptic Children and Caregivers

#### Demographic and Disease Information of Children With Epilepsy

The average age of the children was 4.8 ± 3.18 years, and the average disease duration was 34.2 ± 29.18 months, 47.1% of the children were taking three or more anti-seizure medications, 84.1% could strictly comply with the medical prescription, and 21.1% of the children still had seizures every day. In addition to drug therapy, the proportion of ketogenic diet therapy accounted for 43.2%; 64.9% of the children suffered from comorbid global developmental delay/mental retardation (Supplementary Table 1).

#### Demographic Information of Caregivers of Children With Epilepsy

Of the caregivers of children with epilepsy, 77.9% were mothers, 89% had co-caregivers, and 51.9% wanted financial help; 84.4% of caregivers were satisfied with the physician's explanation of the disease and treatment, but 63.6% felt that they did not have adequate access to education about the disease; 83.7% of the caregivers considered epilepsy to be a terrible disease, but 60.1% were confident in the future of the child, such as control of the disease and living a normal life (Supplementary Table 1).

### Anxiety, Depression Status, Sleep Quality, and Family Functioning of Caregivers in Children With Epilepsy

The average scores of anxiety and depression status and the proportion of abnormal status caregivers of children with epilepsy were high.

Depression status accounted for 65.6% of all caregivers, and anxiety status accounted for 41.9%; 49.0% of the caregivers had poor sleep quality. The scores of family functioning of seven dimensions were from 2.2 to 2.6. The percentage of unhealthy family functioning dimension was from 45.1 to 96.1%, of which the unhealthy behavior control dimension was the highest (96.1%) (Table 1).

**Table 1:** Anxiety and depression status, sleep quality and family functioning of caregivers (*n* = 308).

	**Mean ± SD**	**No. of caregivers**
		**abnormal status, *n* (%)**
SDS standard score	57.5 ± 11.78	202 (65.6)
SAS standard score	48.8 ± 11.27	129 (41.9)
PSQ global score	7.7 ± 3.70	151 (49.0)
FAD: roles	2.53 ± 0.399	222 (72.1)
FAD: communication	2.46 ± 0.399	206 (66.9)
FAD: behavior control	2.45 ± 0.399	296 (96.1)
FAD: problem solving	2.24 ± 0.571	139 (45.1)
FAD: affective responsiveness	2.61 ± 0.476	239 (77.6)
FAD: affective involvement	2.48 ± 0.430	232 (75.3)
FAD: general function	2.37 ± 0.486	244 (79.2)

*SDS, Self-rating Depression Scale; SAS, Self-rating Anxiety Scale; PSQI, Pittsburgh Sleep Quality Index; FAD, Family Assessment Device*.

### The Related Factors of Depression in Caregivers of Children With Epilepsy

Caregivers of children with epilepsy were divided into two groups according to whether they had a depression status or not, and the results were compared between groups (χ^2^ test).

Compared with the non-depression status group, significant differences existed in the following aspects in the depression group: taken anti-seizure medications, ketogenic diet, frequency of seizures, reduced frequency of seizures, and comorbidities of global developmental delay/intellectual disability in their children with epilepsy (*p* < 0.05). The employment status of caregivers, number of co-caregivers, monthly income of families, ability to pay for treatment of epilepsy, attitude toward epilepsy, promising attitude toward the children's future, and desired kinds of assistance were also significantly different between the two groups (*p* < 0.05) (Tables 2, 3).

**Table 2:** Comparison between caregivers without depression status and those with depression status (*n* = 308) (Pearson χ^2^ test) (the general information of caregivers included).

	**No-depression status group, *n* (%)**	**Depression status group, *n* (%)**	**χ^**2**^**	***p***
Relationship			2.597	0.273
Father	26 (24.5)	39 (75.5)		
Mother	80 (19.3)	160 (79.2)		
Employment			7.037	0.030
Full-time work	49 (46.2)	68 (33.7)		
Part-time work	21 (19.8)	34 (16.8)		
Unemployment	36 (34.0)	100 (49.5)		
No. of co-caregivers			16.628	<0.001
0	3 (2.8)	31 (15.3)		
1	40 (37.7)	91 (45.0)		
≥2	63 (59.4)	80 (39.6)		
Educational			1.154	0.764
Junior degree or below	34 (32.1)	69 (34.2)		
High school degree	32 (30.2)	68 (33.7)		
Bachelor's degree	36 (34.0)	60 (29.7)		
Master's degree or above	4 (3.8)	5 (2.5)		
Total income per month (yuan)			18.015	<0.001
≤ 5,000	48 (45.3)	109 (54.0)		
5,000–10,000	32 (30.2)	78 (38.6)		
10,000–20,000	15 (14.2)	10 (5.0)		
≥20,000	11 (10.4)	5 (2.5)		
Medical expenses payment			18.889	<0.001
Able to cover	63 (59.4)	68 (33.7)		
Unable to cover	43 (40.6)	134 (66.3)		
Is epilepsy terrible?			17.436	<0.001
Yes	81 (76.4)	188 (93.1)		
No	25 (23.6)	14 (6.9)		
Promising attitude toward the child future?			12.316	<0.001
Yes	78 (73.6)	107 (53.0)		
No	28 (26.4)	95 (47.0)		
Needed assistance			14.721	0.002
Economic	40 (37.7)	120 (59.4)		
Education for children	37 (34.9)	43 (21.3)		
Knowledge for the epilepsy	23 (21.7)	26 (12.9)		
Other	6 (5.7)	13 (6.4)		

**Table 3:** Comparison between caregivers without depression status and those with depression status (*n* = 308) (Pearson χ^2^ test) (the disease information of children with epilepsy included).

	**No-depression status group, *n* (%)**	**Depression status group, *n* (%)**	**χ^**2**^**	***p***
No. of anti-seizure medications			18.680	<0.001
0	4 (3.8)	3 (1.5)		
1	41 (38.7)	43 (21.3)		
2	28 (26.4)	44 (21.8)		
≥3	33 (31.1)	112 (55.4)		
Seizure frequency			17.800	0.001
Everyday	11 (10.4)	54 (26.7)		
Every week	5 (4.7)	13 (6.4)		
Every month	15 (14.2)	38 (18.8)		
Every year	27 (25.5)	43 (21.3)		
Seizure-free	48 (45.3)	54 (26.7)		
Ketogenic diet treatment			6.804	0.009
Yes	35 (33.0)	98 (48.5)		
No	71 (67.0)	104 (51.5)		
Reduction in the frequency of seizures			13.012	0.005
Total	45 (42.5)	47 (23.3)		
90–99%	18 (17.0)	37 (18.3)		
50–90%	19 (17.9)	48 (23.8)		
<50%	24 (22.6)	70 (34.7)		
With global developmental delay/intellectual disability			9.808	0.002
Yes	56 (53.3)	144 (71.3)		
No	49 (46.7)	58 (28.7)		
With autism spectrum disorder			0.200	0.655
Yes	5 (4.7)	12 (5.9)		
No	101 (95.3)	190 (94.1)		

In the depression status group, proportions of anxiety status, poor sleep quality, unhealthy family functioning dimensions of problem solving, communication, roles, affective involvement, and general function were significantly higher (*p* < 0.05) (Table 4).

**Table 4:** Comparison between caregivers without depression status and those with depression status (*n* = 308) (Pearson χ^2^) (the consequences of scales included).

	**No-depression status group, *n* (%)**	**Depression status group, *n* (%)**	**χ^**2**^**	***p***
Anxiety status			78.282	<0.001
Anxiety status	8 (7.5)	121 (59.9)		
Free of anxiety status	98 (92.5)	81 (40.1)		
Sleep quality			45.022	<0.001
Poor sleep quality	24 (22.6)	127 (62.9)		
Good sleep quality	82 (77.4)	75 (37.1)		
Family functioning of				
Problem solving			18.483	<0.001
Healthy	76 (71.7)	93 (46.0)		
Unhealthy	30 (28.3)	109 (54.0)		
Communication			20.799	<0.001
Health	53 (50.0)	49 (24.3)		
Unhealthy	53 (50.0)	153 (75.7)		
Roles			35.871	<0.001
Healthy	52 (49.1)	34 (16.8)		
Unhealthy	54 (50.9)	168 (83.2)		
Affective responsiveness			14.534	<0.001
Healthy	37 (34.9)	32 (15.8)		
Unhealthy	69 (65.1)	170 (84.2)		
Affective involvement			6.053	0.014
Healthy	35 (33.0)	41 (20.3)		
Unhealthy	71 (67.0)	161 (79.7)		
Behavior control			3.165	0.075
Healthy	7 (6.6)	5 (2.5)		
Unhealthy	99 (93.4)	197 (97.5)		
General function			31.459	<0.001
Healthy	41 (38.7)	23 (11.4)		
Unhealthy	65 (61.3)	179 (88.6)		

*FAD, Family Assessment Device; PSQI, Pittsburgh Sleep Quality Index; SAS, Self-rating Anxiety Scale; SDS, Self-rating Depression Scale*.

### Predictive Factors of Depression Status in Caregivers of Children With Epilepsy (Logistic Regression Analysis)

Factors with significant differences in the comparison of the presence and absence of depression status were included in a binary logistic regression analysis. In the full model containing all predictors: χ^2^ = 1.574, *p* = 0.991 > 0.05, the model correctly classified 85.7% of cases. Without co-caregivers [odds ratio (OR), 5.193], free of anxiety status (OR, 0.063), good sleep quality (OR, 0.446), healthy family role function (OR, 0.344), and healthy family general function (OR, 0.259) were predictors of depression status in caregivers of children with epilepsy (Table 5).

**Table 5:** Predictors of depression status in caregivers (logistic regression analysis).

	***B***	**SE**	**Wald**	***df***	***p***	**Exp (*B*)**	**95% CI**	
							**Lower**	**Upper**
Without co-caregivers	1.647	0.837	3.872	1	0.049	5.193	1.006	26.794
1 co-caregiver	0.159	0.429	0.137	1	0.711	1.172	0.505	2.720
Adverse effect of drugs			8.631	3	0.035			
Ever	−0.205	1.442	0.020	1	0.887	0.814	0.048	13.751
Suffering	0.702	1.486	0.223	1	0.637	2.017	0.110	37.109
Never	−0.859	1.468	0.343	1	0.558	0.423	0.024	7.516
Free of anxiety status	−2.760	0.508	29.517	1	<0.001	0.063	0.023	0.171
Good sleep quality	−0.807	0.403	4.008	1	0.045	0.446	0.203	0.983
Healthy family functioning: roles	−1.068	0.447	5.718	1	0.017	0.344	0.143	0.825
Healthy family functioning: general function	−1.350	0.610	4.902	1	0.027	0.259	0.079	0.857
Constant	3.328	2.183	2.324	1	0.127	27.889		

## Discussion

This study found that anxiety and depression status were common among caregivers of children with epilepsy, with depression status being more prominent. In addition, a considerable proportion of caregivers had poor sleep quality and unhealthy family function. Caregivers' anxiety status, sleep quality, family role dimension, general family function dimension, and the number of co-caregivers were predictors of depression status in caregivers.

It is evident that we included children with epilepsy with more complex conditions. Among the children with epilepsy included in this study, 47.1% took more than three drugs, and 84.1% could strictly abide by the doctor's advice, but 21.1% still had seizures every day. In addition to drug therapy, ketogenic diet therapy was administered to 43.2% of the children; 64.9% suffered from global developmental delay/intellectual disability, probably because our hospital is a national tertiary care epilepsy center (in China, epilepsy centers are classified into levels 1, 2, and 3; level 3 is the most advanced), and children with long-term follow-up visits have more complex situations, and maybe also because caregivers of children with drug-resistant epilepsy visited the hospital frequently and were more willing to participate in the survey.

In this study, the caregivers of children with epilepsy were predominantly mothers, most had co-caregivers, and approximately half of the caregivers wanted financial help. The majority of caregivers were satisfied with the physician's explanation of the disease and treatment, but 63.6% felt that they did not have adequate access to education about the disease; 83.7% of the caregivers considered epilepsy to be a terrible disease, but 60.1% were confident in the future of the child, such as control of disease and living a normal life. As in previous studies, most caregivers were mothers, with a high proportion caring for their children full-time (8). In addition, caregivers of children with epilepsy in this study felt that access to education about the disease was inadequate, which is also consistent with the results of previous studies. Studies conducted in Greece and the United Kingdom showed that caregivers were satisfied with the initial information received from doctors or hospitals about seizures and treatment. However, caregivers often had difficulty to get expertise about epilepsy and needed more access to available social resources and emotional support (17, 18). Parents of children with epilepsy, in Malaysia, also reported a need for epilepsy-related information, ongoing care, and parental support groups (19). The results suggested that we should develop multiple approaches to educate caregivers about epilepsy and help them to seek more social resources, such as community lectures on epilepsy-related disorders and help from social workers or welfare funds.

The mean scores and the proportion of abnormal states were higher among caregivers of children with epilepsy in this study. Depression status accounted for 65.6% of the caregivers, 41.9% of caregivers having anxiety status, and 49.0% having poor sleep quality. The prevalence of depression status in caregivers was much higher than the positive rate of 3.84% for depression in the medical examination population in Shenzhen, China (20). It was also higher than the point prevalence of 3.6% of depression disorders in China's mental health survey (21). The prevalence of anxiety status in caregivers was also much higher than the point prevalence of 5.0% of anxiety disorders in China's mental health survey (21). Our results generally coincide with previous studies that reported proportion of depressive symptoms ranging from 21.6 to 34.9%, anxiety status in 14.5% of caregivers, and poor sleep quality in 37.6% of caregivers (8, 9, 11, 22). There was a higher proportion of depression status, anxiety status, and poorer sleep quality among caregivers in our study. The results might be related to the fact that the caregivers we included were caring for children with drug-resistant epilepsy and that a higher proportion of primary caregivers were mothers. Studies had shown that mothers of children with epilepsy have more anxiety and depression than fathers, mainly related to the frequency and duration of the children's seizures (22).

This study also found that the proportion of unhealthy family functioning of caregivers of children with epilepsy ranged from 45.1 to 96.1%, and the highest of which was the behavioral control function (96.1%). The primary function of the family is to provide specific environmental conditions for the healthy development of the physical, psychological, and social aspects of family members. The failure of the family to achieve its essential functions can easily lead to various problems in family members (23). This study suggested that unhealthy family functioning was common in the families of children with complex epilepsy, and the caregivers needed help at the family level.

There were significant differences in the aspects of the severity of epilepsy, the attitude toward epilepsy, the number of co-caregivers, the employment of the caregivers, and so on, when they were divided into groups according to whether they had a depression status or not. There were also significant differences in anxiety status, poor sleep quality, and unhealthy family function in the depression status group. Based on the results above, binary logistic regression analysis showed that anxiety status, poor sleep quality, unhealthy family role function, and unhealthy family general family function increased the prevalence of caregiver depression status. The presence of co-caregivers (≥1) decreased the incidence of caregiver depression status.

Our results showed that the severity of the children's disease was not a predictor of caregivers' depression status. Previous studies had shown that the frequency, severity, and unpredictability of seizures brought a burden on families (12). The degree of anxiety and depression of the child's mother was mainly related to the frequency and duration of the child's seizures (24). This difference might result from that our subjects were caregivers of childhood epilepsy and the high proportion of children with drug-resistant epilepsy. On this basis, group comparisons were made based on the presence or absence of depression status in the caregivers, and then the predictors of caregiver depression were analyzed, resulting in a finding that the child's illness was not significant in predicting the caregiver's depression status.

A Danish study investigating the mental status and stress levels of parents of children with severe epilepsy showed that the increased caregiver stress was associated with the younger age of the child, a higher level of the child's difficulties such as emotional and behavioral problems, and social difficulties. Seizure type, seizure frequency, epilepsy category, and age at seizure onset were not significantly associated with the level of the caregiver's stress. Caregivers' social support and the experience of having control over life circumstances were associated with a lower level of caregivers' stress. When all related variables were analyzed by standard multiple regression analysis, only the child's difficulties, caregivers' control of their own situation, and social support were significant predictors of caregivers' stress level (8). Another study in Qilu Hospital of Shandong University in China also showed that family resilience explained 3.5 and 14.9% of sleep quality and depression. The better the family resilience, the better the quality of sleep and the less the depression among caregivers. Interventions to improve family resilience may enhance sleep quality, reduce depression, and improve family parenting of children with epilepsy (11).

Similarly, this study also suggested that caregivers' own status and family functioning were predictors of the caregiver's depression status. And this is the first time to investigate family function among caregivers of childhood epilepsy in China. As shown in the study, the clinical staff should help caregivers to identify their family problems and assist them to improve the family function.

Although we tried our best to expand our sample size, this was a single-center study. In addition, this study used a web-based questionnaire, which was completed by the respondents individually. Some participants could not obtain timely instructions on how to fill out the questionnaire, and some information was not filled out in a standard manner, which might affect the accuracy of some responses. Therefore, we chose the widely used self-assessment scales to minimize bias.

## Conclusion

Anxiety and depression were prevalent among caregivers of children with epilepsy, with depression status being more prominent. In addition, a significant proportion of caregivers had poor sleep quality and unhealthy family functioning. The caregiver's own anxiety status and sleep quality, family role function and general function, and the number of co-caregivers were predictors of caregiver depression status. In clinical practice, the above situation should be addressed alongside the treatment of children with epilepsy.

## Data Availability Statement

The original contributions presented in the study are included in the article/Supplementary Material, further inquiries can be directed to the corresponding authors.

## Ethics Statement

The studies involving human participants were reviewed and approved by Shenzhen Children's Hospital. Written informed consent for participation was not required for this study in accordance with the national legislation and the institutional requirements.

## Author Contributions

MeZ conceived of the analysis. HZ, MaZ, YF, SH, and JH contributed to the data collection and analysis. MeZ and HZ wrote the first draft of the manuscript. JL provided critical feedback on the first draft. MeZ, HZ, and JL managed the production process. All authors read and approved the final manuscript.

## Funding

This study was supported by the Shenzhen Fund for Guangdong Provincial Highlevel Clinical Key Specialties (No. SZGSP012).

## Conflict of Interest

The authors declare that the research was conducted in the absence of any commercial or financial relationships that could be construed as a potential conflict of interest.

## Publisher's Note

All claims expressed in this article are solely those of the authors and do not necessarily represent those of their affiliated organizations, or those of the publisher, the editors and the reviewers. Any product that may be evaluated in this article, or claim that may be made by its manufacturer, is not guaranteed or endorsed by the publisher.
