# Supplementary material for: Investigation of Anxiety, Depression, Sleep, and Family Function in Caregivers of Children With Epilepsy
Source: Front Neurol. 2021 Oct 25;12:744017. doi: 10.3389/fneur.2021.744017 (PMC8575681; doi:10.3389/fneur.2021.744017)
Supplement: Supplementary file 1 [file Table_1.docx]

Table 1 The demographic characteristics and disease information of children with epilepsy（N=308）

N (%)

| age of the children (years old) |  |
| --- | --- |
| ≤3 | 110(35.7) |
| 3＜age≤6 | 105(34.1) |
| 6＜age≤12 | 93(30.2) |
| Gender |  |
| Boy | 172(55.8) |
| Girl | 136(44.2) |
| The only child in the family |  |
| Yes | 115(37.3) |
| No | 193(62.7) |
| Number of anti-epilepsy drugs |  |
| 0 | 7(2.3) |
| 1 | 84(27.3) |
| 2 | 72(23.4) |
| ≥3 | 145(47.1) |
| Seizure frequency |  |
| everyday | 65(21.1) |
| Every week | 18(5.8) |
| Every month | 53(17.2) |
| Every year | 70(22.7) |
| Seizure free | 102(33.1) |
| Seizure type |  |
| Focal seizures | 139(45.1) |
| Generalized seizures | 79(25.6) |
| Unknown and unclassified | 90(29.2) |
| Drug compliance |  |
| Strictly | 259 (84.1) |
| Good | 39(12.7) |
| General | 4(1.3) |
| Terribly | 1(0.3) |
| Without drug | 5(1.6) |
| Ketogenic diet treatment |  |
| Yes | 133(43.2) |
| No | 175(56.8) |
| Surgical treatment |  |
| Yes | 21(6.8) |
| No | 287(93.2) |
| Vagus nerve stimulation treatment |  |
| Yes | 7(2.3) |
| No | 301(97.7) |
| With global developmental delay/Intellectual disability  Yes  No  absence | 200(64.9)  107(34.7)  1(0.3) |
| With Autism spectrum disorder |  |
| Yes | 17(5.5) |
| No | 291(94.5) |

Table 2 Demographic information of caregivers of children with epilepsy

|  | N（%） |
| --- | --- |
| Relationship with the child |  |
| Father | 65 (21.1) |
| Mother | 240 (77.9) |
| Other | 3 (1.0) |
| Gender |  |
| Female | 66 (21.4) |
| Male | 242 (78.6) |
| The primary caregiver of the child |  |
| Yes | 259 (84.1) |
| No | 49 (15.9) |
| Employment |  |
| Full-time work | 117 (38.0) |
| Part-time work | 55 (17.9) |
| Unemployment | 136 (44.2) |
| The number of co-caregivers |  |
| 0 | 34 (11.0) |
| 1 | 131 (42.5) |
| ≥2 | 143 (46.4) |
| Education |  |
| Master's degree or above | 9 (2.9) |
| Bachelor’s degree | 96 (31.2) |
| Highschool | 100 (32.5) |
| Junior high school or below | 103 (33.4) |
| The medical staff's explanation of the child's condition |  |
| Totally understandable | 79 (25.6) |
| Almost understandable | 181 (58.8) |
| Not very clear | 45 (14.6) |
| Not clear at all | 3 (1.0) |
| Enough access to obtain information on epilepsy |  |
| Yes | 112 (36.4) |
| No | 196 (63.6) |
| Is epilepsy terrible |  |
| Yes | 269 (87.3) |
| No | 39 (12.7) |
| Promising attitude towards the child's future |  |
| Yes | 185 (60.1) |
| No | 123 (39.9) |
| Needed assistance |  |
| Economic | 160 (51.9) |
| General education for children | 80 (26.0) |
| Knowledge for the epilepsy | 49 (15.9) |
| Other | 19 (6.2) |
